# Clinical Risk Factors and High-Risk Plaques in Coronary Computed Tomography

**DOI:** 10.3390/diseases14070228

**Published:** 2026-06-25

**Authors:** Piotr Żarczyński, Patrycja Brzóska-Ritter, Maciej Haberka

**Affiliations:** Department of Cardiology, School of Health Sciences, Medical University of Silesia, 40-635 Katowice, Poland; patrycja.brzoska1@poczta.onet.eu (P.B.-R.);

**Keywords:** CCT, HRP, CAD-RADS, cardiovascular risk stratification, CAD

## Abstract

**Background:** Cardiovascular (CV) risk estimation is usually based on the assessment of classic risk factors and the extent of coronary artery stenosis. However, a substantial rate of acute coronary syndromes (ACS) and sudden cardiac deaths (SCD) is observed in patients with high-risk atherosclerotic plaques (HRP), even in the absence of significant stenosis. Therefore, this study aimed to evaluate the predictive value of traditional clinical risk factors for the presence of HRP in patients scheduled for coronary computed tomography (CCT). **Methods:** This single-center study included 123 patients undergoing CCT for suspected coronary artery disease (CAD). Atherosclerotic plaque morphology (HRP) and the degree of coronary artery stenosis (CAD-RADS categories) were assessed in all the patients. CV risk factors, including LDL serum levels and CT Calcium score (CS), were analyzed. **Results:** The study cohort was mostly males (54.5%), with an average age of 60.40 ± 12.45 years and typical risk factors: hypertension (70%), diabetes (22%), obesity (30%), and smoking (20%). Most patients (88%) were found to have coronary atherosclerosis with nonobstructive disease (CAD-RADS 1–2) in 39% of patients. HRP was confirmed in over one-fifth of the participants (22%), with half of the patients in the CAD-RADS 2 category. There were no differences in CV risk factors between patients with and without HRP in CCT. No significant clinical predictor of HRP in CCT was identified. **Conclusions:** CV risk factors do not predict HRP in CCT, which may underestimate the real risk of ACS and SCD.

## 1. Introduction

Cardiovascular diseases, including coronary artery disease, remain the leading cause of death in developed countries [1]. Although cardiac diagnostics have relied primarily on assessing the hemodynamic significance of stenoses, contemporary evidence indicates that the development of acute coronary syndrome (ACS) is driven both by plaque instability and the degree of vessel stenosis [2]. The sudden rupture of atherosclerotic plaque that initiates the coagulation cascade results in acute thrombosis and a subsequent restriction of coronary blood flow that forms the pathogenetic basis for ACS and sudden cardiac death [3]. Furthermore, autopsy studies have revealed that most deaths due to ACS are precipitated by the rupture of an atherosclerotic plaque that had not previously caused significant vascular narrowing, underscoring the importance of plaque instability over luminal narrowing [4,5].

Coronary computed tomography (CCT) has significantly advanced noninvasive coronary artery disease diagnostics, currently serving as the first-line imaging modality in patients with suspected chronic coronary syndromes [6]. Beyond its strong negative predictive value for excluding significant stenoses, a major advantage of CCT is the ability to simultaneously assess vessel architecture and atherosclerotic plaque morphology, including the identification of high-risk plaques (HRP). The presence of these vulnerable plaques is associated with a high risk of rupture and subsequent ACS. Typical CCT-derived features of HRP include a low-attenuation, positive vessel remodeling, spotty calcifications, and the “napkin-ring” sign [7,8].

In parallel with advances in cardiovascular imaging, artificial intelligence (AI) is increasingly being explored as a supportive technology in healthcare. In medicine, AI-based approaches have been applied across several domains, including medical image segmentation, computer-aided diagnosis, disease risk prediction, workflow optimization, and personalized treatment planning. In imaging-based diagnostics, AI may facilitate automated lesion detection, quantitative image analysis, and extraction of clinically relevant imaging biomarkers [9]. Although AI-based image analysis was not used in the present study, these developments highlight the broader trend toward quantitative and reproducible imaging-based risk assessment.

The clinical significance of HRP identification is profound, as it shifts the therapeutic paradigm from merely treating coronary artery stenosis to actively preventing myocardial infarction. Patients diagnosed with HRP face a disproportionately higher risk of major adverse cardiovascular events (MACE) and require effective and timely pharmacological interventions, including dual antiplatelet therapy or high-dose statins, even in the absence of hemodynamically significant coronary artery stenosis [7,10].

While the impact of classic risk factors on the development of atherosclerosis is well-evidenced [11], the relationship between these factors and the presence of a specific unstable atherosclerotic plaque phenotype remains poorly understood, posing a significant diagnostic and therapeutic challenge [12]. Despite the effectiveness of CCT in identifying HRP, it cannot be routinely utilized as a population-wide screening test due to limitations regarding ionizing radiation, contrast administration, and lack of widespread availability. Therefore, a significant research gap exists: the absence of a readily available, rapid, and precise method for identifying individuals harboring HRPs prior to resorting to advanced imaging techniques [13]. It remains unclear why some individuals with typical risk factors develop stable, calcified atherosclerotic plaques, while others with similar risk profiles exhibit highly vulnerable plaque morphologies prone to precipitating ACS.

Therefore, this study aimed to assess the associations between clinical characteristics and the presence of HRP in CCT.

## 2. Materials and Methods

### 2.1. Study Group

All consecutive patients scheduled for CCT at the Upper Silesian Medical Center in Katowice were screened for eligibility (March 2025–May 2026), and finally, 125 patients were included in the study group. These outpatients were suspected of having coronary artery disease based on their symptoms and/or medical history. Two individuals were subsequently excluded from the study due to imaging artifacts (*n* = 123).

The inclusion criteria included the age between 20 and 80 years and written informed consent to participate in the study. The exclusion criteria were as follows: endocarditis, myocarditis, pericarditis, or systemic vasculitis within the 6 months preceding the study; stroke or transient ischemic attack within the 2 months prior to the study; acute or chronic inflammatory conditions or autoimmune diseases; infectious diseases within the last month; acute or chronic clinically significant kidney disease (estimated glomerular filtration rate [eGFR] < 30 mL/min/1.73m^2^) or liver disease; thyroid dysfunction (hypo- or hyperthyroidism); malignant neoplasms, lymphomas, or leukemias diagnosed and/or treated within the 2 years prior to study enrollment; and a history of alcohol or illicit substance abuse within 1 year prior to enrollment.

### 2.2. Data Collection

All clinical data, including comorbidities, clinical symptoms, their severity, and concomitant pharmacotherapy, were obtained from electronic medical records and standardized patient interviews.

The CCT was performed using a third-generation dual-source CT scanner (Siemens Somatom Force; Siemens Healthineers, Erlangen, Germany) in the high-volume cardiovascular imaging center. The CCT scans and Calcium Score (CS) assessment were performed according to the current guidelines [14,15]. The image acquisition of the coronary arteries was obtained in 1.5- and 0.6-mm slice reconstructions, both before (calcium score protocol) and after intravenous administration of the radiocontrast agent (Ultravist 370, Bayer AG, Berlin, Germany). Sublingual nitroglycerin and intravenous metoprolol (if needed) were used in all patients prior to the examination to achieve optimal conditions and image quality. The CS was presented using Agatston Units (AU). The CAD-RADS classification and plaque morphology assessment were performed according to current CAD Reporting and Data System guidelines [16]. HRP was defined according to CAD-RADS 2.0 as the presence of at least two high-risk plaque features within a coronary atherosclerotic plaque. The assessed HRP features included low-attenuation plaque, positive remodeling, spotty calcification, and the napkin-ring sign.

Routine laboratory tests assessing the lipid profile, peripheral blood count, fasting blood glucose, and creatinine levels were performed within a month prior to the CCTA examination in all patients.

### 2.3. Statistical Analysis

The Shapiro–Wilk test was used to assess the normality of data distribution. Continuous variables were compared using the Student’s *t*-test or the nonparametric Mann–Whitney U-test, depending on the distribution. Categorical variables were compared using the Chi-square test or Fisher’s exact test, as appropriate. The Pearson correlation coefficient and the nonparametric Spearman’s rank correlation coefficient were used to determine relationships between continuous variables. Furthermore, univariate and multivariate logistic regression models were employed to identify independent predictors of HRP. Receiver operating characteristic (ROC) analysis and the area under the curve (AUC) were calculated to assess the diagnostic performance of the investigated continuous parameters. A *p*-value < 0.05 was considered statistically significant. All analyses were performed using Medcalc software (version 23.5, Ostend, Belgium). Sex-stratified comparisons of lipid parameters were performed post hoc as exploratory analyses. These analyses were not prespecified as primary study objectives and were, therefore, considered hypothesis-generating. Due to the limited sample size, the results were interpreted with caution.

## 3. Results

### 3.1. Baseline Characteristics

A total of 123 patients with CCT due to suspected CAD were finally included in the study group. The study cohort comprised mostly males (54.5%) with an average age of 60.40 ± 12.45 years and cardiovascular risk factors as follows: hypertension (70%), diabetes (22%), obesity (30%), and smoking (20%) (Table 1).

### 3.2. Coronary Artery Disease and High-Risk Atherosclerotic Plaques

The severity of coronary artery disease was assessed in all the study patients. In the study group, 12.0% of patients had coronary arteries without atherosclerotic plaques (CAD-RADS 0), 39.3% had nonobstructive disease (CAD-RADS 1–2), 23.9% exhibited moderate stenosis (CAD-RADS 3), and 24.8% presented with a severe stenosis or total occlusion (CAD-RADS 4–5).

High-risk atherosclerotic plaques (HRP) were identified in 27 individuals, representing 22% of the study population. In the HRP (+) subgroup (n = 27), there were 16 males (59%), the prevalence of diabetes was 22%, and 26% of patients were smokers. The mean BMI in the HRP (+) cohort was 29.6 ± 4.6 kg/m^2^. The mean CS in the HRP (+) group was 188 ± 244 AU, compared with 239 ± 515 AU in the HRP (−) group, which did not reach statistical significance (*p* = 0.2). Both subgroups usually showed a few coronary plaques; half of the patients had at least one coronary stenosis (>50%), and one third showed a one-vessel disease. However, there were no differences between the HRP (+) and HRP (−) groups in clinical characteristics, lipid profile, fasting glucose, CAD parameters, or HRP-related pharmacotherapy (Table 2).

**Figure 1:**
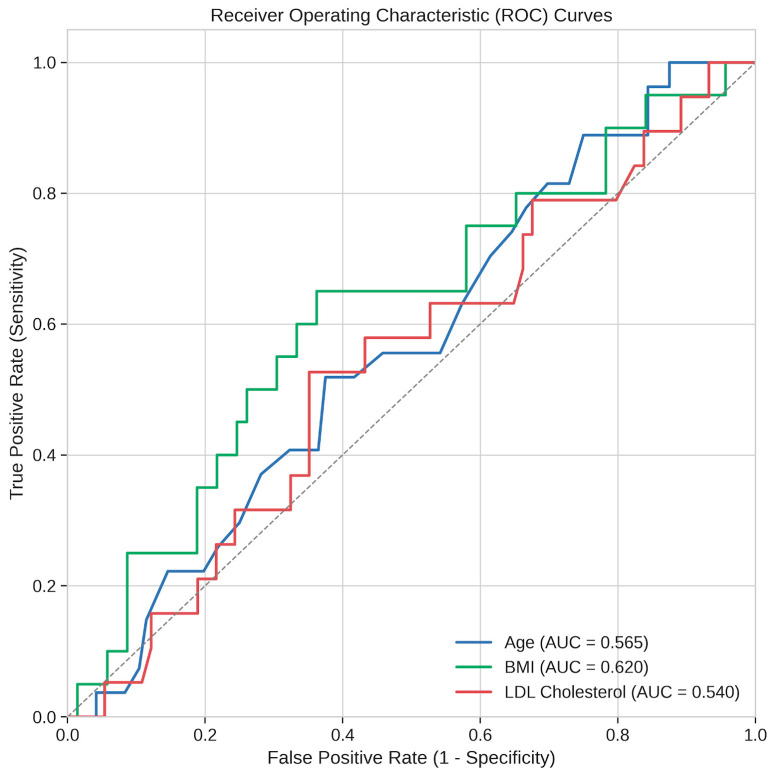
Receiver operating characteristic (ROC) curves for age, body mass index (BMI), and LDL cholesterol predicting the occurrence of high-risk atherosclerotic plaque (HRP).

Based on CT findings, most HRP (+) patients (55.6%) were classified as CAD-RADS 2, while the remaining individuals in this subgroup were found to have the following CAD-RADS categories: 3 (29.6%), 4 (7.4%), or 5 (7.4%). Figure 1 presents the rates of HRP, sex, mean age, and mean LDL concentrations across the CAD-RADS categories. The highest rate of patients with HRP was in the CAD-RADS 2 group (mostly females), with a mean age of 62.4 ± 5 years and a mean LDL of 95.9 mg/dL. Notably, the highest LDL levels were observed in the lower CAD-RADS categories, likely reflecting a lack of prior intensive lipid-lowering therapy in these patients (Table 2).

There was no difference in the presence of HRP between males and females (23.9% vs. 19.6%, *p* = 0.66). In a post hoc exploratory, hypothesis generating sex-stratified analysis of lipid parameters, women showed higher HDL cholesterol (59.2 ± 17.9 vs. 48.2 ± 18.2 mg/dL; *p* = 0.006), LDL cholesterol (135.6 ± 100.1 vs. 96.8 ± 53.8 mg/dL; *p* = 0.02), and total cholesterol (212.17 ± 111.5 vs. 169.43 ± 67.1 mg/dL; *p* = 0.025), whereas triglyceride levels were higher in men than in women (144.4 ± 92.7 vs. 107.9 ± 46.8 mg/dL; *p* = 0.02). Because this analysis was not prespecified and the number of patients within each sex stratum was limited, these findings should be interpreted with caution and considered hypothesis generating only.

### 3.3. Association Analysis and Prediction Models

A comprehensive analysis of potential risk factors associated with HRP was performed. Pearson correlation analysis revealed a significant positive association between the number of atherosclerotic plaques and age (r = 0.5; *p* < 0.0001) or CS (r = 0.5; *p* < 0.0001). As expected, the number of coronary plaques was associated with CS (r = 0.74, *p* < 0.0001). None of the parameters showed a statistically significant association with the HRP, including age (*p* = 0.3), BMI (*p* = 0.13) and the number of plaques (*p* = 0.28). Furthermore, a multivariate model with age, LDL, BMI, and HDL failed to achieve statistical significance (*p* = 0.8) for HRP plaques.

ROC curve analysis confirmed that the studied parameters had poor discriminatory performance and lacked acceptable clinical predictive value for HRP, with areas under the curve (AUC) of 0.56 for age, 0.62 for BMI, and 0.54 for LDL cholesterol, all close to chance-level discrimination (Figure 1).

The detailed diagnostic performance of these parameters is summarized in Table 3.

A post-hoc power analysis demonstrated that, for correlation analyses performed in the whole cohort (N = 123), the study achieved a statistical power of over 85% to detect a moderate correlation coefficient of r = 0.30 at α = 0.05. Therefore, the correlation analyses were adequately powered to detect moderate associations. However, the HRP (+) subgroup included only 27 patients and was markedly smaller than the HRP (−) subgroup (27 vs. 96). Consequently, between-group comparisons and ROC curve analyses were underpowered to detect small-to-moderate effects.

**Figure 2:**
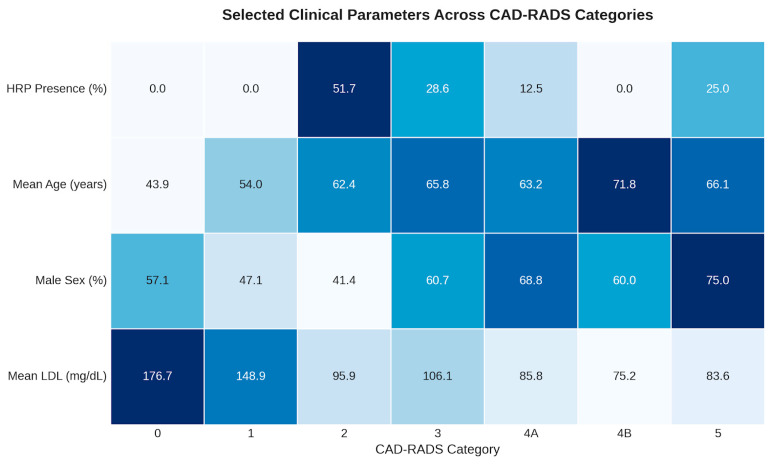
Heatmap demonstrating the distribution of selected clinical and laboratory parameters across CAD-RADS categories. To account for the varying units of measurement, color intensity was normalized row-wise. The darkest shade in each row represents the highest mean value or percentage for that specific parameter across the CAD-RADS spectrum.

The distribution of selected clinical and laboratory parameters across CAD-RADS categories was assessed (Figure 2).

### 3.4. Pharmacotherapy

Finally, the study evaluated the association of concomitant pharmacotherapy, specifically focusing on antiplatelet agents and hypolipidemic drugs (statins and ezetimibe). The rates of ezetimibe (*p* = 0.45) and antiplatelet (*p* = 1.0) use were not significantly different between the HRP (+) and (−) groups. However, a weak negative association between statin dose and the presence of HRP was observed, which revealed a trend for statistical significance (r = −0.16, *p* = 0.07).

## 4. Discussion

Our single-center study performed in the reference cardiovascular center provides novel results important for clinical practice. The main finding was that more than one-fifth of the patients scheduled for CCTA due to suspected CAD were found to have HRP within the coronary arteries. However, we found that clinical characteristics are not associated with HRP, suggesting an independent and added value of CCT.

The clinical significance of HRP is very important for cardiovascular risk and clinical prognosis. It is well-evidenced that HRPs are prone to spontaneous rupture, which triggers the initiation of the coagulation cascade, leading to acute thrombosis, ACS and sudden cardiac death (SCD) [17,18]. We showed that none of the cardiovascular risk factors, including age, sex and LDL serum levels, or CT CS, were associated with HRP. Moreover, age, BMI, and LDL serum levels showed very poor discriminatory ability for HRP, with AUC values close to chance, indicating that these variables cannot be considered clinically useful standalone predictors of HRP. The above results suggest that cardiovascular risk is underestimated in more than one-fifth of the cardiovascular outpatients. Previous studies have also shown that laboratory markers and traditional risk factors are weakly associated with coronary artery disease in CCT [18,19]. However, the research evidence and clinical value for HRP have increased recently. Moreover, the development of CCT scanners has significantly improved the quality of images, which are essential for HRP images. Therefore, we present novel results, which are based on a modern CT scanner with high-quality images.

In our cohort, neither an apparently acceptable lipid profile nor the absence of advanced CAD excluded HRP, as over half of HRP (+) individuals were classified as CAD-RADS 2. Therefore, qualitative plaque assessment may complement stenosis-based evaluation and conventional risk-factor assessment.

The clinical importance of HRP identification has been well-evidenced in large clinical trials, including PROMISE and SCOT-HEART [10,19]. The presence of HRP was shown to be a stronger predictor of future cardiovascular events compared to the assessment of the degree of coronary stenosis alone [7,8,19]. Our results support these observations, as a significant proportion of patients with HRP were diagnosed with atherosclerotic plaques, which were not associated with significant stenosis (CAD-RADS 2). This presents a significant diagnostic challenge, as the high-risk patient remains hidden behind a stable clinical picture. Although classic risk factors such as diabetes, smoking, and hypertension are very important for the long-term development of atherosclerosis and coronary heart disease, our data confirm that they are not sufficient to identify patients at the highest risk for plaque rupture. Several studies have been conducted to develop a model for predicting the presence of HRP based on blood biomarkers and clinical findings, but none of them was sensitive enough to replace CCT [20,21]. At the invasive level, optical coherence tomography (OCT) represents a complementary tool for plaque characterization, providing high-resolution assessment of plaque morphology and mechanisms of plaque destabilization [22]. However, because OCT is an invasive technique, it should be considered complementary to, rather than a substitute for, noninvasive CCT-based risk stratification. While trials using high-sensitivity CRP (hs-CRP) and lipid subfractions have shown their associations with the presence of HRP, the feasibility of their routine use is low, mainly due to a lack of specificity and the inability to distinguish systemic from local inflammation [23]. We found very low predictive values for age, LDL serum levels and BMI, which suggest that even strong traditional risk factors do not rule out HRP in CCT. It suggests that morphological transformation of a stable plaque into a high-risk phenotype is a complex phenomenon that cannot be detected with standard laboratory tests. This finding strengthens the position of CCT not only as a secondary diagnostic tool but also as an independent modality capable of providing incremental prognostic value that goes beyond classical risk stratification.

In our study, the distribution of LDL levels among CAD-RADS categories was particularly noteworthy. Paradoxically, patients in the lower categories (CAD-RADS 0–1) demonstrated higher mean LDL concentrations compared to patients with more advanced CAD. This phenomenon is explained in our cohort by the fact that patients with traditional risk factors and earlier cardiological screening in outpatients usually receive earlier and more intensive hypolipidemic therapy with statins. Given that hypercholesterolemia is a well-known CV risk factor with a very low LDL target level [11,24], the great majority of patients scheduled for CCT use statins. This lack of native and off-medicine LDL serum levels makes the clinical characteristic even more complicated in clinical practice. However, hypercholesterolemia, including Familial Hypercholesterolemia, is a well-known risk factor for noncalcified atherosclerotic plaques [25]. On the other hand, in patients with early-stage atherosclerosis, drug therapy may not have been initiated yet, leading to higher LDL levels despite lower degrees of stenosis. This may partly explain why single-time-point LDL concentrations showed limited predictive value for HRP in the present cohort.

Our analysis of pharmacotherapy deliberately focused on ASA and hypolipidemic treatment, as ASA and statins are the main drug classes with a well-evidenced effect on HRP. The PARADIGM clinical study clearly demonstrated that statin therapy promotes plaque stabilization by significantly reducing low-attenuation plaque volume and increasing its calcification [26,27]. In the present cohort, statin therapy was less frequent in the HRP(+) group than in the HRP(−) group (48.1% vs. 67.7%), although this difference did not reach statistical significance. This trend may be clinically relevant, as the absence of statin therapy or insufficient statin intensity could contribute to persistence of a lipid-rich, noncalcified, and potentially vulnerable plaque phenotype. Therefore, HRP presence may be influenced not only by LDL concentration measured at the time of CCT but also by previous exposure to lipid-lowering therapy, treatment duration, adherence, and statin dose intensity. This is particularly important because single-time-point LDL values may not fully reflect cumulative atherogenic exposure or the stabilizing effect of long-term pharmacotherapy. Furthermore, ASA is crucial in preventing atherothrombotic events following HRP rupture. Therefore, the association between the use of these drugs and the presence of HRP was considered [28,29]. More broadly, our findings should be interpreted within the evolving framework of multimodality imaging in cardiovascular prevention. A recent comprehensive review emphasized that imaging modalities, including coronary artery calcium scoring, echocardiography, CCTA, cardiac magnetic resonance, and emerging imaging-derived markers, may complement traditional risk stratification in both primary and secondary prevention. In this context, CCT-based plaque morphology assessment may provide additional information beyond stenosis severity and conventional cardiovascular risk factors [30].

Our study has some limitations. First, the relatively small sample size (n = 123), which may have limited the identification of small associations between clinical data and the presence of HRP. This limitation is particularly important for analyses comparing HRP (+) and nHRP (−) patients, because the HRP (+) subgroup included only 27 individuals and was substantially smaller than the HRP (−) subgroup. No a priori power calculation was performed before study initiation. Although the post-hoc power analysis indicated adequate power to detect moderate correlations in the whole cohort, subgroup comparisons and ROC curve analyses were underpowered to detect small-to-moderate effects. Therefore, null findings from these analyses should be interpreted cautiously and cannot exclude weaker associations between clinical variables and HRP. Second, the sex-stratified lipid analysis was performed post-hoc and was not pre-specified; therefore, given the limited sample size within each sex stratum, these findings should be considered exploratory and hypothesis-generating only. Furthermore, this was a cross-sectional study without long-term follow-up to assess a link between HRP and clinical events. Third, this was a cross-sectional study in patients with various pharmacotherapies, which could also affect coronary plaque morphology. However, this was a study of cardiovascular patients in a real-life setting.

In summary, our study indicates that patient clinical profiles and routine laboratory markers have limited ability to predict the presence of HRP in CCT. Similarly, stenosis severity assessed by CAD-RADS did not reliably reflect plaque vulnerability. Therefore, plaque morphology assessment may provide additional information for cardiovascular risk stratification and personalized preventive pharmacotherapy. Further studies should focus on the development of novel CT scanners fit for early screening and new drug molecules aimed at stabilizing atherosclerotic plaques.

## 5. Conclusions

High-risk plaques (HRP) were detected in more than one-fifth of patients (21.95%) referred for CCTA due to suspected coronary artery disease.

Traditional risk factors and routine laboratory tests demonstrated a low predictive value and do not allow for the reliable identification of patients with HRP in coronary vessels.

Most patients with HRP fell into the CAD-RADS 2 category; therefore, assessing risk solely based on the degree of vascular stenosis leads to an underestimation of cardiovascular risk in patients with a stable clinical picture.

The qualitative assessment of atherosclerotic plaque morphology using CCTA serves as an independent diagnostic tool that goes beyond traditional cardiovascular risk assessment methods. This is crucial for implementing early, personalized preventive pharmacotherapy.

## Figures and Tables

**Table 1:** Clinical characteristics of the study group.

Parameter	Mean ± SD, Median [IQR], or n (%)
Age (years)	62.00 [52.50–70.00]
Sex (Male)	67 (54.5%)
BMI (kg/m^2^)	28.17 ± 4.76
Hypertension	84 (70.0%)
Obesity	37 (30%)
Diabetes mellitus	27 (22.5%)
Active smoking	24 (19.8%)
LDL (mg/dL)	89.00 [64.00–141.00]
HDL (mg/dL)	49.00 [40.00–62.00]
TG (mg/dL)	111.00 [80.00–154.00]

Data are presented as mean ± standard deviation (SD) for continuous variables with a normal distribution (BMI) or median [interquartile range, IQR] for continuous variables with a nonnormal distribution (Age, LDL, HDL, and TG), and as number (percentage) for categorical variables. BMI: Body Mass Index; LDL: Low-Density Lipoprotein; HDL: High-Density Lipoprotein; TG: Triglycerides.

**Table 2:** Comparison of clinical and coronary artery disease characteristics in patients with and without high-risk plaque (HRP).

Parameter	HRP (−) (n = 96)	HRP (+) (n = 27)	*p*-Value
*Demographics & Anthropometrics*			
Age (years)	59.8 ± 13	62.7 ± 10.3	0.31
Male sex, n (%)	51 (53.1%)	16 (59.3%)	0.73
BMI (kg/m^2^)	27.8 ± 4.7	29.6 ± 4.6	0.12
*Comorbidities & Clinical Presentation*			
Current smoking, n (%)	17 (17.7%)	7 (28.0%)	0.39
Diabetes mellitus, n (%)	21 (21.9%)	7(25.9%)	0.96
Hypertension, n (%)	67 (69.8%)	17 (70.8%)	1.0
NYHA class > I, n (%)	21 (21.9%)	10 (37%)	0.18
CCS class > I, n (%)	23 (24%)	9 (33.3%)	0.46
*Laboratory Findings*			
Total Cholesterol (mg/dL)	189.2 ± 97.6	185.8 ± 65.8	0.65
LDL Cholesterol (mg/dL)	114.0 ± 84.2	113.7 ± 60.2	0.6
HDL Cholesterol (mg/dL)	53.4 ± 16.9	52.2 ± 25.3	0.23
Triglycerides (mg/dL)	128.4 ± 82	124.3 ± 54.1	0.76
Fasting glucose (mg/dL)	113.3 ± 38.6	109.6 ± 16.3	0.63
*Coronary Artery Disease*			
Calcium Score (Agatston units)	254.4 ± 573.7	188.1 ± 244	0.21
Number of plaques	3.9 ± 3.5	4.7 ± 2.7	0.08
Stenosis > 50%	48 (50%)	12 (44.4%)	0.77
Stenosis > 70%	25 (26.0%)	4 (14.81%)	0.27
Stenosis in 1 coronary artery	26 (27.08%)	9 (33.33%)	0.58
Stenosis in 2 coronary arteries	16 (16.67%)	2 (7.41%)	
Stenosis in 3 coronary arteries	6 (6.25%)	1 (3.70%)	
*Pharmacotherapy*			
Statin therapy (any dose), n (%)	65 (67.7%)	13 (48.1%)	0.10
Ezetimibe therapy, n (%)	27 (28.1%)	5 (18.5%)	0.45
Antiplatelet therapy (ASA), n (%)	30 (31.2%)	9 (33.3%)	1.0

Data are presented as mean ± standard deviation (SD) for continuous variables and as number (percentage) for categorical variables. Statistical significance between the HRP (−) and HRP (+) groups was assessed using the Mann–Whitney U-test for continuous data and the Chi-square test (or Fisher’s exact test, as appropriate) for categorical variables. A *p*-value of <0.05 was considered statistically significant. “Stenosis > 50%” and “Stenosis > 70%” indicate the number of patients with at least one coronary artery stenosis exceeding the respective threshold. “Stenosis in 1, 2, or 3 coronary arteries” indicates the number of patients with >50% stenosis involving one, two, or three coronary arteries, respectively. HRP: High-Risk Plaque; BMI: Body Mass Index; NYHA: New York Heart Association; CCS: Canadian Cardiovascular Society; LDL: Low-Density Lipoprotein; HDL: High-Density Lipoprotein; ASA: Acetylsalicylic Acid.

**Table 3:** Diagnostic performance of selected clinical parameters in predicting the presence of High-Risk Plaque (HRP).

Parameter	Sensitivity (%)	Specificity (%)	PPV (%)	NPV (%)
Age	51.9	62.5	28.0	82.2
BMI	65.0	63.8	34.2	86.3
LDL	52.6	64.9	27.08	84.2

Positive Predictive Value (PPV), Negative Predictive Value (NPV), Body Mass Index (BMI), Low-Density Lipoprotein (LDL).

## Data Availability

Data is unavailable due to privacy of study participants.

## Abbreviations

The following abbreviations are used in this manuscript:

ACS	Acute Coronary Syndrome
AI	artificial intelligence
ASA	Acetylsalicylic Acid
AUC	Area Under the Curve
BMI	Body Mass Index
CAD	Coronary Artery Disease
CAD-RADS	Coronary Artery Disease Reporting and Data System
CCTA	Coronary Computed Tomography Angiography
CS	Calcium Score
CT	Computed Tomography
HDL	High-Density Lipoprotein
HRP	High-Risk Plaque
hs-CRP	High-Sensitivity C-Reactive Protein
IQR	Interquartile Range
LDL	Low-Density Lipoprotein
MACE	Major Adverse Cardiovascular Events
NPV	Negative Predictive Value
NYHA	New York Heart Association
PPV	Positive Predictive Value
ROC	Receiver Operating Characteristic
SCD	Sudden Cardiac Death
SD	Standard Deviation
TG	Triglycerides

## References

[1] Timmis A., Townsend N., Gale C.P., Torbica A., Lettino M., Petersen S.E., Mossialos E.A., Maggioni A.P., Kazakiewicz D., May H.T. (2020). European Society of Cardiology: Cardiovascular Disease Statistics 2019. Eur. Heart J..

[2] Libby P., Pasterkamp G. (2015). Requiem for the “Vulnerable Plaque”. Eur. Heart J..

[3] Libby P. (2013). Mechanisms of Acute Coronary Syndromes and Their Implications for Therapy. N. Engl. J. Med..

[4] Virmani R., Burke A.P., Farb A., Kolodgie F.D. (2006). Pathology of the Vulnerable Plaque. J. Am. Coll. Cardiol..

[5] Arbab-Zadeh A., Fuster V. (2015). The Myth of the “Vulnerable Plaque”: Transitioning from a Focus on Individual Lesions to Atherosclerotic Disease Burden for Coronary Artery Disease Risk Assessment. J. Am. Coll. Cardiol..

[6] Neumann F.J., Sechtem U., Banning A.P., Bonaros N., Bueno H., Bugiardini R., Chieffo A., Crea F., Czerny M., Delgado V. (2020). 2019 ESC Guidelines for the Diagnosis and Management of Chronic Coronary Syndromes: The Task Force for the Diagnosis and Management of Chronic Coronary Syndromes of the European Society of Cardiology (ESC). Eur. Heart J..

[7] Puchner S.B., Liu T., Mayrhofer T., Truong Q.A., Lee H., Fleg J.L., Nagurney J.T., Udelson J.E., Hoffmann U., Ferencik M. (2014). High-Risk Plaque Detected on Coronary CT Angiography Predicts Acute Coronary Syndromes Independent of Significant Stenosis in Acute Chest Pain: Results from the ROMICAT-II Trial. J. Am. Coll. Cardiol..

[8] Motoyama S., Ito H., Sarai M., Kondo T., Kawai H., Nagahara Y., Harigaya H., Kan S., Anno H., Takahashi H. (2015). Plaque Characterization by Coronary Computed Tomography Angiography and the Likelihood of Acute Coronary Events in Mid-Term Follow-Up. J. Am. Coll. Cardiol..

[9] Porkar P., Mehrabipour F., Pourasad M.H., Movassagh A.A., Nazari K., Ghaderzadeh M., Gheisari M., Salehnasab C., Almasi S. (2025). Enhancing Cancer Zone Diagnosis in MRI Images: A Novel SOM Neural Network Approach with Block Processing in the Presence of Noise. Iran. J. Blood Cancer.

[10] Williams M.C., Moss A.J., Dweck M., Adamson P.D., Alam S., Hunter A., Shah A.S.V., Pawade T., Weir-McCall J.R., Roditi G. (2019). Coronary Artery Plaque Characteristics Associated with Adverse Outcomes in the SCOT-HEART Study. J. Am. Coll. Cardiol..

[11] Mach F., Baigent C., Catapano A.L., Koskinas K.C., Casula M., Badimon L., Chapman M.J., De Backer G.G., Delgado V., Ference B.A. (2020). 2019 ESC/EAS Guidelines for the Management of Dyslipidaemias: *Lipid Modification to Reduce Cardiovascular Risk*: The Task Force for the Management of Dyslipidaemias of the European Society of Cardiology (ESC) and European Atherosclerosis Society (EAS). Eur. Heart J..

[12] Williams M.C., Kwiecinski J., Doris M., McElhinney P., D’Souza M.S., Cadet S., Adamson P.D., Moss A.J., Alam S., Hunter A. (2020). Low-Attenuation Noncalcified Plaque on Coronary Computed Tomography Angiography Predicts Myocardial Infarction: Results from the Multicenter SCOT-HEART Trial (Scottish Computed Tomography of the HEART). Circulation.

[13] Chang H.J., Lin F.Y., Lee S.E., Andreini D., Bax J., Cademartiri F., Chinnaiyan K., Chow B.J.W., Conte E., Cury R.C. (2018). Coronary Atherosclerotic Precursors of Acute Coronary Syndromes. J. Am. Coll. Cardiol..

[14] Abbara S., Blanke P., Maroules C.D., Cheezum M., Choi A.D., Han B.K., Marwan M., Naoum C., Norgaard B.L., Rubinshtein R. (2016). SCCT Guidelines for the Performance and Acquisition of Coronary Computed Tomographic Angiography: A Report of the Society of Cardiovascular Computed Tomography Guidelines Committee: Endorsed by the North American Society for Cardiovascular Imaging (NASCI). J. Cardiovasc. Comput. Tomogr..

[15] Hecht H.S., Cronin P., Blaha M.J., Budoff M.J., Kazerooni E.A., Narula J., Yankelevitz D., Abbara S. (2017). 2016 SCCT/STR Guidelines for Coronary Artery Calcium Scoring of Noncontrast Noncardiac Chest CT Scans: A Report of the Society of Cardiovascular Computed Tomography and Society of Thoracic Radiology. J. Cardiovasc. Comput. Tomogr..

[16] Cury R.C., Leipsic J., Abbara S., Achenbach S., Berman D., Bittencourt M., Budoff M., Chinnaiyan K., Choi A.D., Ghoshhajra B. (2022). CAD-RADS^TM^ 2.0—2022 Coronary Artery Disease-Reporting and Data System: An Expert Consensus Document of the Society of Cardiovascular Computed Tomography (SCCT), the American College of Cardiology (ACC), the American College of Radiology (ACR), and the North America Society of Cardiovascular Imaging (NASCI). Radiol. Cardiothorac. Imaging.

[17] Motoyama S., Sarai M., Harigaya H., Anno H., Inoue K., Hara T., Naruse H., Ishii J., Hishida H., Wong N.D. (2009). Computed Tomographic Angiography Characteristics of Atherosclerotic Plaques Subsequently Resulting in Acute Coronary Syndrome. J. Am. Coll. Cardiol..

[18] Narula J., Nakano M., Virmani R., Kolodgie F.D., Petersen R., Newcomb R., Malik S., Fuster V., Finn A.V. (2013). Histopathologic Characteristics of Atherosclerotic Coronary Disease and Implications of the Findings for the Invasive and Noninvasive Detection of Vulnerable Plaques. J. Am. Coll. Cardiol..

[19] Ferencik M., Mayrhofer T., Bittner D.O., Emami H., Puchner S.B., Lu M.T., Meyersohn N.M., Ivanov A.V., Adami E.C., Patel M.R. (2018). Use of High-Risk Coronary Atherosclerotic Plaque Detection for Risk Stratification of Patients with Stable Chest Pain: A Secondary Analysis of the PROMISE Randomized Clinical Trial. JAMA Cardiol..

[20] Conte E., Andreini D., Magnoni M., Masson S., Mushtaq S., Berti S., Canestrari M., Casolo G., Gabrielli D., Latini R. (2021). Association of High-Risk Coronary Atherosclerosis at CCTA with Clinical and Circulating Biomarkers: Insight from CAPIRE Study. J. Cardiovasc. Comput. Tomogr..

[21] Annink M.E., Kraaijenhof J.M., Beverloo C.Y.Y., Oostveen R.F., Verberne H.J., Stroes E.S.G., Nurmohamed N.S. (2025). Estimating Inflammatory Risk in Atherosclerotic Cardiovascular Disease: Plaque over Plasma?. Eur. Heart J. Cardiovasc. Imaging.

[22] Buonpane A., Trimarchi G., Di Muro F.M., Nardi G., Ciardetti M., Coceani M.A., Pastormerlo L.E., Paradossi U., Berti S., Trani C. (2025). From Vision to Illumination: The Promethean Journey of Optical Coherence Tomography in Cardiology. J. Clin. Med..

[23] Crea F., Liuzzo G. (2013). Pathogenesis of Acute Coronary Syndromes. J. Am. Coll. Cardiol..

[24] Visseren F., Mach F., Smulders Y.M., Carballo D., Koskinas K.C., Bäck M., Benetos A., Biffi A., Boavida J.M., Capodanno D. (2021). 2021 ESC Guidelines on Cardiovascular Disease Prevention in Clinical Practice. Eur. Heart J..

[25] Miname M.H., Bittencourt M.S., Moraes S.R., Alves R.I.M., Silva P.R.S., Jannes C.E., Pereira A.C., Krieger J.E., Nasir K., Santos R.D. (2019). Coronary Artery Calcium and Cardiovascular Events in Patients with Familial Hypercholesterolemia Receiving Standard Lipid-Lowering Therapy. JACC Cardiovasc. Imaging.

[26] Lee S.E., Chang H.J., Sung J.M., Park H.B., Heo R., Rizvi A., Lin F.Y., Kumar A., Hadamitzky M., Kim Y.J. (2018). Effects of Statins on Coronary Atherosclerotic Plaques: The PARADIGM Study. JACC Cardiovasc. Imaging.

[27] Van Rosendael A.R., Van Den Hoogen I.J., Gianni U., Ma X., Tantawy S.W., Bax A.M., Lu Y., Andreini D., Al-Mallah M.H., Budoff M.J. (2021). Association of Statin Treatment with Progression of Coronary Atherosclerotic Plaque Composition. JAMA Cardiol..

[28] Casado-Arroyo R., Bayrak F., Sarkozy A., Chierchia G.B., De Asmundis C., Brugada P. (2012). Role of ASA in the Primary and Secondary Prevention of Cardiovascular Events. Best Pract. Res. Clin. Gastroenterol..

[29] Gargiulo G., Windecker S., Vranckx P., Gibson C.M., Mehran R., Valgimigli M. (2016). A Critical Appraisal of Aspirin in Secondary Prevention: Is Less More?. Circulation.

[30] Carerj M.L., Restelli D., Poleggi C., Di Bella G., Zito C., Manganaro R., Piccione M.C., Trimarchi G., Farina A., Micari A. (2025). The Role of Imaging in Cardiovascular Prevention: A Comprehensive Review. J. Cardiovasc. Echogr..

